# The Association of Matrix Metalloproteinases with Chronic Kidney Disease and Peripheral Vascular Disease: A Light at the End of the Tunnel?

**DOI:** 10.3390/biom10010154

**Published:** 2020-01-17

**Authors:** Michele Provenzano, Michele Andreucci, Carlo Garofalo, Teresa Faga, Ashour Michael, Nicola Ielapi, Raffaele Grande, Paolo Sapienza, Stefano de Franciscis, Pasquale Mastroroberto, Raffaele Serra

**Affiliations:** 1 Department of Health Sciences, Renal Unit, “Magna Graecia” University, 88100 Catanzaro, Italy; michiprov@hotmail.it (M.P.); andreucci@unicz.it (M.A.); teresa_faga@yahoo.it (T.F.); ashourmichael@yahoo.com (A.M.); 2 Division of Nephrology, University of Campania “Luigi Vanvitelli”, 80100 Naples, Italy; carlo.garofalo@hotmail.it; 3 Interuniversity Center of Phlebolymphology (CIFL), “Magna Graecia” University, 88100 Catanzaro, Italy; infermierenicola@hotmail.it (N.I.); defranci@unicz.it (S.d.F.); 4 Department of Public Health and Infectious Disease, “Sapienza” University of Rome, 00185 Rome, Italy; 5 Department of Radiology, Vibo Valentia Hospital, 89900 Vibo Valentia, Italy; 6 Department of Surgery “P. Valdoni”, “Sapienza” University of Rome, 00161 Rome, Italy; raffaele.grandeprospero@gmail.com (R.G.); paolo.sapienza@uniroma1.it (P.S.); 7 Department of Medical and Surgical Sciences, “Magna Graecia” University, 88100 Catanzaro, Italy; 8 Department of Experimental and Clinical Medicine, “Magna Graecia” University, 88100 Catanzaro, Italy; mastroroberto@unicz.it

**Keywords:** metalloproteinases, MMPs, TIMPs, CKD, peripheral vascular disease, biomarkers, proteinuria, eGFR, PAD.

## Abstract

Chronic Kidney Disease (CKD) represents a risk factor for fatal and nonfatal cardiovascular (CV) events, including peripheral vascular disease (PVD). This occurs because CKD encompasses several factors that lead to poor prognoses, mainly due to a reduction of the estimated glomerular filtration rate (eGFR), the presence of proteinuria, and the uremic inflammatory milieu. The matrix metalloproteinases (MMPs) are a group of zinc-containing endopeptidases implicated in extracellular matrix (ECM) remodeling, a systemic process in tissue homeostasis. MMPs play an important role in cell differentiation, angiogenesis, inflammation, and vascular damage. Our aim was to review the published evidence regarding the association between MMPs, PVD, and CKD to find possible common pathophysiological mechanisms. MMPs favor ECM deposition through the glomeruli, and start the shedding of cellular junctions and epithelial-mesenchymal transition in the renal tubules. MMP-2 and -9 have also been associated with the presence of systemic vascular damage, since they exert a pro-inflammatory and proatherosclerotic actions. An imbalance of MMPs was found in the context of PVD, where MMPs are predictors of poor prognoses in patients who underwent lower extremity revascularization. MMP circulating levels are increased in both conditions, i.e., that of CKD and PVD. A possible pathogenic link between these conditions is represented by the enhanced production of transforming growth factor-β that worsens vascular calcifications and atherosclerosis and the development of proteinuria in patients with increased levels of MMPs. Proteinuria has been recognized as a marker of systemic vascular damage, and this may explain in part the increase in CV risk that is manifest in patients with CKD and PVD. In conclusion, MMPs can be considered a useful tool by which to stratify CV risk in patients with CKD and PVD. Further studies are needed to investigate the causal-relationships between MMPs, CKD, and PVD, and to optimize their prognostic and predictive (in response to treatments) roles.

## 1. Introduction

Chronic Kidney Disease (CKD) is defined as the presence of abnormalities in kidney function or structure for at least 3 months [1]. The Kidney Disease: Improving Global Outcomes (KDIGO) guidelines classify CKD according to the level of estimated glomerular filtration rate (eGFR), a marker of kidney function, and the amount of urine protein (proteinuria or albuminuria), which represents the principal marker of kidney damage and the primary cause of CKD [2]. The onset of CKD exerts a deleterious impact on individual health. Indeed, it has been demonstrated that either an eGFR reduction < 60 mL/min/1.73 m^2^ or a small increase in proteinuria are associated with a poor prognosis, as shown by the increased rate of cardiovascular (CV) fatal and nonfatal events, all-cause mortality, and the progression of kidney disease resulting in the need for renal replacement therapies (kidney transplantation or dialysis) [3,4].

CV risk is severely increased in patients with CKD, and the impact of CV events in this population is crucial if one considers that the rate of CV events (such as myocardial infarction, stroke, arrhythmias, peripheral vascular disease, and chronic heart failure) over time is higher than the risk for kidney disease progression [5]. This strong association has been attributed to the coexistence of traditional and nontraditional CV risk factors in CKD patients, with the former being represented by hypertension, smoking, hypercholesterolemia, and metabolic abnormalities, and the latter by the two main prognostic measures of CKD, i.e., proteinuria and eGFR [6,7].

Among the wide spectrum of CV events, a relevant role is portrayed by peripheral vascular disease (PVD). It has been demonstrated that the presence of mild-to-moderate CKD increases the risk of peripheral artery disease, leg revascularization, leg amputation, and hospitalizations [8]. Either an eGFR reduction below 60 mL/min/1.73 m^2^ or slight increases of albuminuria (>30 mg/g) have been associated with a 1.5 to 4 times higher risk of peripheral artery disease (PAD). This evidence is strong, being derived from patients without PAD at basal visit, and reproducible, being confirmed in the general population, as well as in high-risk populations, regardless of the geographic area. Notably, the rate of PVD among patients with End-Stage Kidney Disease (ESKD) is higher than the incidence of acute myocardial infarction, stroke, and arrhythmias [9]. Hence, CKD patients warrant clinical surveillance and prompt the need for strategies to prevent the onset of PVD.

The magnitude of the association is so important that research has recently focused on discovering new biomarkers that are potentially useful in the clinical management of CKD patients at increased risk for PVD. Matrix Metalloproteinases (MMPs) are zinc-containing endopeptidases that are involved in regulating tissue development and homeostasis [10]. Although all MMPs are better acknowledged for their role in remodeling the extracellular matrix (ECM), they actually interact with both ECM and non-ECM substrates. Cell adhesion molecules and growth factors or their receptors represent these latter group of substrates. The wide range of interactions in which MMPs play an active role also explains why these endopeptidases participate in a number of functions such as cell differentiation, migration, apoptosis, and angiogenesis. On the other hand, MMPs have also been attributed to a profibrotic and pro-inflammatory role. MMP-2 and MMP-7 were increased in plasma and urine samples of CKD patients, and may affect direct damage to the kidney with the onset of albuminuria [11]. Moreover, imbalances in the expression and the levels of MMPs or their inhibitors have been linked to the structural changes that occur in the development of PAD, atherosclerotic plaque maturation, and arterial remodeling [12].

The purpose of this review is to examine the role of MMPs in increasing the risk of peripheral vascular disease by the specific aggravating condition of Chronic Kidney Disease.

## 2. Materials and Methods

The PubMed and ISI Web of science databases were searched for articles by using the following terms: ‘chronic kidney disease”, “chronic renal insufficiency”, “metalloproteinases”, “MMP” “atherosclerosis” “peripheral vascular disease”. Titles and abstracts were screened by three authors (Michele Provenzano, Michele Andreucci, and Raffaele Serra) to identify potentially relevant studies. All potentially eligible studies were subsequently evaluated in detail by one reviewer and three authors (Michele Provenzano, Michele Andreucci, Carlo Garofalo, and Raffaele Serra) through consideration of the full text. Reference lists of retrieved articles were also searched for relevant publications. Clinical trial, meta-analyses, narrative review, and systematic reviews published in the last 10 years were included. Bibliographies of relevant articles and reviews were manually screened to identify additional studies. Studies were excluded if they were not in the English language, if they did not fit the research question, or if they had insufficient data.

## 3. Results

### 3.1. Study Selection

Initial database searches yielded 180 studies from PubMed and 445 from ISI Web of Science in the last 10 years. After the evaluation of the bibliographies of the relevant articles, we evaluated 67 eligible full text articles. The current evidence on MMP expression and kidney disease, the link between MMPs and CV risk specifically in CKD patients, and the association between MMPs and peripheral vascular disease are described below.

### 3.2. Metalloproteinases and the Kidney

**Figure 1:**
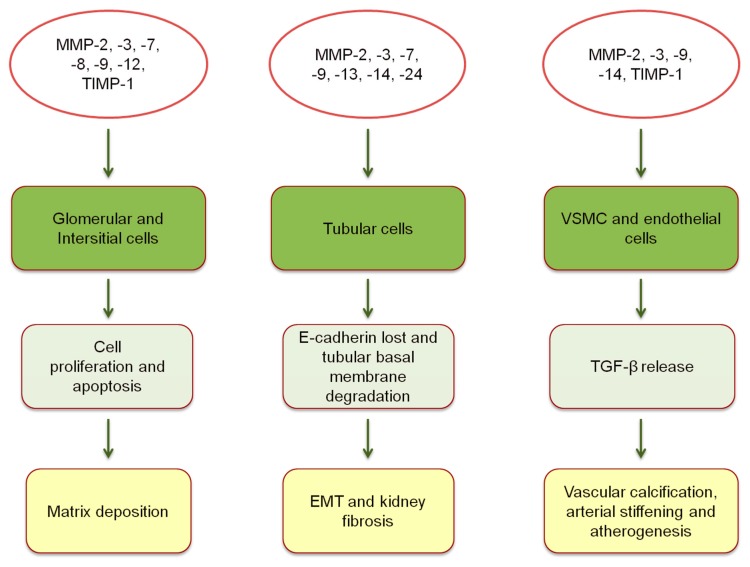
Expression of MMPs and TIMPs, and pathophysiological mechanisms of vascular and kidney damage [10,11,27,28,29,30,31]. EMT, epithelial-mesenchymal transition; VSMC, vascular smooth muscle cells; TGF-β, transforming growth factor-β.

MMPs are classified, according to their structure (or function) and the substrate selectivity, into six groups: Collagenases (MMP-1, MMP-8 and MMP-13), which cleave native collagen and with possible antifibrotic function; Gelatinases (MMP-2 and MMP-9), whose function is to cleave denatured collagens, type IV collagens in basement membranes and some chemokines; Stromelysins (MMP-3, MMP-10, MMP-11 and MMP-19), which degrade a number of substances such as fibronectin, laminin but are unable to cleave native collagen; Matrilysins (MMP-7 and MMP-26) act by degrading ECM components (laminin and entactin); Membrane-type MMPs (MMP-14, -15, -16, -17, -24, and -25), so called because they are anchored to the exterior of the cell membrane; and other MMPs that are tissue or cell-type specific [10,13]. The MMP activity is modulated by a series of four known enzymes called tissue inhibitors of metalloproteinases (TIMPs). TIMPs participate either in the activation or inhibition of MMP activity and, like MMPs, regulate several cellular functions such as cell proliferation, apoptosis, and angiogenesis [14]. It has been demonstrated that MMPs exert a role in the development of proteinuric kidney diseases in humans. Indeed, a number of studies depicted increased serum and urine levels of MMP-2, -8, and -9 in diabetic patients, with MMP-9 in particular being positively correlated with the degree of proteinuria in these patients [15,16,17,18]. It is remarkable that, in addition to MMP-9, urinary levels of neutrophil gelatinase-associated lipocalin (NGAL) have been found to increase in patients with diabetic nephropathy [19]. NGAL and MMP-9 are coexpressed, and their interaction prevents the degradation of MMP-9. It has, thus, been postulated that the increase in NGAL may prolong the action of MMP-9 as a trigger of kidney damage [20]. MMP-7, which is normally expressed in the proximal and distal convoluted tubules, as well as in the collecting duct, is found to be overexpressed in diabetic patients where it is also inversely correlated with the degree of kidney function [21]. Other than in diabetic patients, MMP expression is altered in many other glomerular diseases. Typical patterns of MMP-2 and MMP-9 are differentially expressed in patients with focal segmental glomerulosclerosis, minimal change disease, membranous nephropathy, and ANCA-associated vasculitis [22,23,24,25,26]. Regardless of the specific kidney disease involved, several mechanisms of damage have been put forward to explain the pathophysiologic effects of MMPs (Figure 1).

MMPs intervene in all phases of renal fibrosis, from infiltration of mononuclear cells to cells proliferation and scarring. All these processes lead to a progressive decline of renal function in CKD patients. Henger and Colleagues performed a hierarchical clustering analysis to assess the differential gene expression in human kidney fibrosis. Interestingly, they observed that several MMP (MMP-3, -13, -14) genes were upregulated in different degrees of fibrosis [32]. With respect to inflammation, different MMPs play different, often opposing, actions. MMP-7 and MMP-9 expand inflammatory processes, particularly by their chemotactic effect on human dendritic cells [33,34]. Conversely, MMP-13 and MMP-14 have been hypothesized to act as anti-inflammatory mediators [35]. MMP-3 may also promote epithelial-to-mesenchymal transition, the conversion of tissue phenotype, from the epithelial to fibroblastic, thereby accelerating fibrosis [36]. Abnormalities in the accumulation/degradation of ECM due to imbalanced levels of MMPs and TIMPs have also been described in rat and humans [37,38,39]. Indeed, downregulation of MMP-1 and the overexpression of TIMP-1, MMP-2, MMP-7, and MMP-9 are associated with a profibrotic effect, as well as with a destructive effect on renal parenchyma [38,39]. Moreover, increased TIMP-1 plasma levels were predictors of incident CKD, regardless of other systemic and inflammatory biomarkers (C-Reactive Protein or Brain Natriuretic Peptide) and many clinical parameters (liver function, concomitant lipid-lowering, or antihypertensive medications) [40].

### 3.3. Vascular Effects of Metalloproteinases in CKD Patients

**Figure 2:**
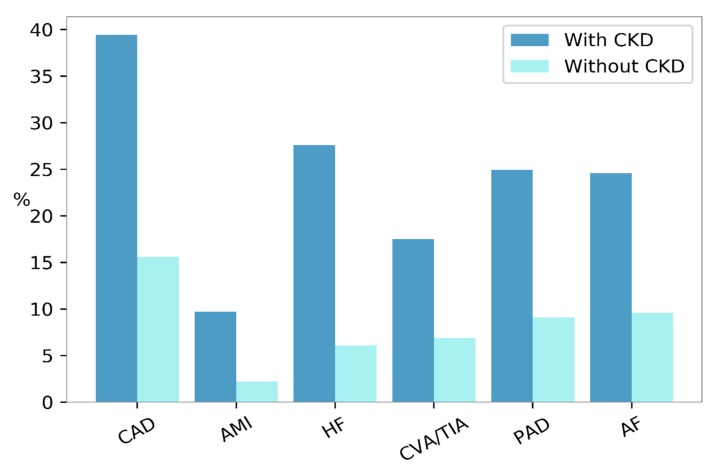
Prevalence of cardiovascular diseases according to the presence (dark blue bars) or absence (turquoise bars) of Chronic Kidney Disease (CKD) in the United States, in the year 2015. AF, atrial fibrillation; AMI, acute myocardial infarction; CAD, coronary artery disease; CVA/TIA, cerebrovascular accident/transient ischemic attack; HF, heart failure; PAD, peripheral arterial disease.

The structural remodeling of ECM, together with the profibrotic effect of MMPs, are detrimental for other organs apart from the kidney. Elevated blood concentrations of TIMP-1 have been associated with an increased risk of developing chronic heart failure (CHF) and, in patients already diagnosed with CHF, they were predictors of poor prognoses [41,42]. Moreover, the increase in MMP-9 and TIMP-1 conferred risk of all-cause mortality and incident CV disease in community studies [43,44]. Possible explanations for the relationship between MMPs and CV risk are varied. Whereas all MMPs are likely risk factors for atherosclerosis and cardiac dysfunction, a more specific mechanism of damage has been postulated for MMP-9 and TIMP-1. Indeed, MMP-9 is involved in the intracellular cleavage of myosin filaments, a mechanism that leads to ventricular hypertrophy [45]. TIMP-1 has shown a direct relation with the left ventricular mass in the Framingham Heart Study participants [41]. The CKD condition is associated with an increased prevalence of CV morbidity and mortality. The United States Renal Data System (USRDS) showed that the frequency of each CVD, including myocardial infarction, coronary artery disease, and peripheral vascular disease was higher among patients with CKD compared with those without (Figure 2) [9].

The CV burden in these patients is significant if one considers that the rate of CV events is similar to that of reaching ESKD [46,47]. As a result of this epidemiological and public health evidence, great effort has been placed in finding early atherosclerosis biomarkers that predict CV events in CKD patients and serve as a possible target for new therapeutic agents [48]. MMPs were included in the set of investigated substances. Indeed, MMP-2 was positively associated with carotid Intima-Media Thickness (cIMT) and abdominal aortic calcification, suggesting that an association between this MMP and subclinical atherosclerosis is plausible [49,50,51]. Moreover, MMP-2 was also found to be higher in patients with a positive history of CV disease vs no history of CV disease [52,53]. MMP-9 was also strongly associated with cIMT, the development of carotid plaques, and systemic atherosclerosis [49,50]. MMP deregulation is intensified in patients with advanced CKD stages that are also associated with a reduction of their clearance. All these mechanisms enhance the inflammatory process that is chronically activated in CKD patients, due to oxidative stress, the uremic milieu, and the metabolic acidosis [54]. The combination of uremic status and imbalance in pro-inflammatory substances (such as MMPs) accelerates the atherosclerotic process, arterial stiffness, and vascular calcification, and impairs the vascular repair process as well [55]. Circulating MMP-2, -9, and -10 have been found to increase in CKD patients, and have been implicated in the vascular damage process. Moreover, MMP-2 and -9 are able to reduce the plaque stability in advanced CKD stages, thus rendering the plaque itself more prone to rupture [56,57].

### 3.4. Metalloproteinases and Peripheral Vascular Disease

MMPs play a role in the pathogenesis and prognosis of arterial and venous disease. With respect to the damage occurring in the arterial tissue, multiple studies have shown that MMPs are involved in one or more steps of atherogenesis and aneurysm development [58]. Evidence that confirms this hypothesis comes from both basic and clinical studies. During the progression of atherosclerotic plaques, a number of MMPs are produced, including TIMP-1, MMP-1, -2, -3, -9, and -14 [59]. An in vivo study on Fischer male rats showed that TIMP-1 directly regulates the smooth muscle cell migration [27]. A similar function has been recognized in human studies on MMP-14 and TIMP-1, with being both involved in the process of cellular migration to the plaque fibrous cap and plaque inflammation [28,29]. MMP-9 also contributes to the destruction of the fibrous cap itself in patients with increased CV risk [30]. Interestingly, increased concentrations of MMP-2, -3, and -9 have been found within the aneurysm human tissue, being mainly produced by the macrophages localized in the aneurysm wall [31]. These different enzymes are differentially expressed according to the aneurysm dimensions and severity [60,61]. MMP-2 is increased in small aneurysms (<5.5 cm), whereas MMP-9 is dominant in large aneurysms (5.5–7 cm). Moreover, it has also been shown that different localizations of MMP activity within the aneurysm wall modify the risk of rupture [60]. In clinical studies, abnormal circulating levels of MMP-1, -2, -8, -9, and TIMP-1 were found in patients with Peripheral Arterial Disease; their increase was attributed to the presence of ischemic tissue [12,62,63]. Circulating levels of MMP-1 and -8 were also found as predictors of poor prognosis, in terms of major amputation or death, in patients who underwent lower extremity bypass [64]. These multiple findings enhanced the importance of MMPs as biomarkers of arterial disease severity that also provide important prognostic information in clinical practice.

Regarding venous diseases, it has been observed that alterations in ECM remodeling are common in the case of varicose veins and chronic venous insufficiency (CVI). With regards to varicose veins, the expression of MMP-1, -2, -3, -7, and -9 is increased, particularly in the smooth muscular cell of the vein wall, both in human and mice models [65,66]. Moreover, an analysis of human saphenous vein showed that this expression is even higher in varicose veins with inflammation, as compared to those without inflammation [65]. Mechanisms underlying the association between MMPs and varicose vein physiopathology might involve the effect of MMPs on ECM degradation and the relaxation of the venous wall [66]. An upregulation of MMP-1, -2, -9, and -13, together with a downregulation of TIMP-1 and TIMP-2, have been described in patients with CVI [67,68]. The distribution of MMP varies based upon the stage (from CVI to active wound) and cells, suggesting that MMP-1 and TIMP-1 are needed in the re-epithelialization phase, while MMP-9 and -13 primarily participate in the remodeling of the collagenous matrix [69]. A summary of the principal pieces of evidence provided from this paper is depicted in Table 1.

### 3.5. Synthetic Metalloproteinases Inhibitors in Experimental and Clinical Research

MMP activity is regulated at different levels, including either intracellular (mRNA expression and post-translational modification of MMP structure) or extracellular (stimulation or inhibition of their enzymatic activity from endogenous or exogenous substrates) process. The net activity of MMPs is a crucial step, since its up- or down- regulation could affect MMP activity, and ultimately lead to metabolic diseases, cancer, cardiovascular, and renal disorders [72]. For these reasons, new pharmacological agents that interfere with MMP activity have been developed and utilized as potential tools that could benefit a wide spectrum of patients. Synthetic MMPs inhibitors (MMPs-I) include broad-spectrum and specific MMPs-I. The vast majority of these compounds contain Zn^2+^ in their structure, and have structured as Zn^2+^ binding globulin (ZBG). Indeed, ZBGs inactivate MMPs by displacing the Zn^2+^- bound water in the MMPs, and favor the anchorage of the drug to the MMPs substrate binding-pocket [73]. ZBGs encompass hydroxamic acids Batimastat (BB-94), Marimastat (BB-2516), and Ilomastat (GM6001), that displays a broad-spectrum inhibition of MMPs. More selective ZBGs molecules have also been developed, and include hydrazides and sulfonylhydrazides that specifically inhibit MMP-1, -2, and -9. Hydroxamic ZBGs are effective, but they have poor oral bioavailability and, by inhibiting multiple MMPs, cause several musculoskeletal side effects [74]. Hence, heterocyclic bidentate chelators have been developed that have shown more biostability and lower toxicity in cells assays. Tetracyclines are antibiotic molecules that, by chelating Zn^2+^ ion, are able to inhibit MMPs. Doxycycline inhibits MMP-2 and-9 [75]. Chemically-modified tetracyclines reach higher plasma levels for prolonged periods of time, require less frequent administration, are associated with lower rate of side effects when administered orally, and are thus preferred over conventional tetracyclines [76]. Apart from zinc-based compounds, several MMPs-I act by a noncompetitive, nonzinc-binding, mechanism of inhibition. They show high selectivity that minimizes the side effects, and thus, are considered very promising molecules [77]. MMPs-Is have already been used in pilot studies in mice and in human models of kidney damage [11]. MMPs-I BB-1101 has shown to reduce proteinuria in rats with anti-Thy1.1 nephritis, an experimental model of glomerular damage induced by antibody against Thy 1 gene [78]. A similar effect on proteinuria was found with the MMPs-I BB-94 in an experimental model of kidney allograft rejection in mice and the tetracycline antibiotic doxycycline in human patients with diabetic nephropathy already under renin-angiotensin-aldosterone inhibition [79,80]. Interestingly, doxycycline also reduced the aneurysm expansion in small randomized clinical trials enrolling patients with abdominal aortic aneurysm [81,82].

## 4. Discussion

The evidence for how the circulating levels or the expression of MMPs increase cardiovascular risk is well documented in both basic and clinical studies. However, to our knowledge, this represents the first review to encapsulate and describe the dual association between MMPs and CKD and MMPs and PVD.

Two large meta-analyses have separately summarized the strength of the relationship between MMP-2, TIMP-1, and subclinical atherosclerosis in CKD patients or between MMPs and vascular risk, regardless of the level of kidney function [58,83]. They concluded that imbalances in MMPs/TIMPS were implicated in the pathogenesis, clinical manifestations, and prognosis of arterial and venous diseases.

According to our results, we can also affirm that the CKD condition contributes to reinforcing the risk-pathways oriented from MMPs toward PVD. Numerous pieces of evidence support this: (1) Numerous MMP molecules are likely responsible for both CV (including PVD) and kidney damage and related clinical manifestations. Hence, such biomarkers warrant further investigation. The Gelatinases MMP-2 and MMP-9 are produced by both intrinsic glomerular and tubular cells [52,70]. It has been shown that the increased activity of MMP-2 and MMP-9 across the kidney tubules may lead to structural alterations in the tubular basement membrane, which starts epithelial-mesenchymal transition (EMT). EMT consists of the process in which a component of adherent junctions, the E-cadherin, and cell adhesion are mislaid, whereas epithelial cells acquire a mesenchymal phenotype by expressing and producing α-smooth muscle actin (αSMA) and matrix protein. All these mechanisms subsequently trigger tubular atrophy and fibrosis [71,84]. Moreover, Peiskerova and coworkers have demonstrated that levels of MMP-2 were significantly higher in CKD patients Stage III–V as compared to those with Stage I–II, and correlated with fibroblast growth factor 23 (FGF-23) and levels of serum phosphate, two important surrogates of oxidative stress and CV risk in these patients [52,71]. Similarly, gelatinases have also been implicated in the onset of structural changes associated with PAD. Circulating levels of MMP-2 and MMP-9 have been found to increase in patients with acute and chronic lower limb ischemia (i.e., intermittent claudication and critical ischemia), as well as in patients with hyperlipidemia when compared with healthy subjects [12,85,86]. The connecting link between CKD, gelatinases, and PAD is mainly represented by the increased atherosclerotic risk and arterial remodeling due to the imbalance of ECM enzymes. Indeed, MMP-2 and MMP-9 are capable of releasing latent transforming growth factor beta (TGF-β), which acts as a mediator in the cross-talk between endothelial cells and vascular smooth muscle cells [71]. Imbalances in MMPs and alterations in endothelial cells are a direct causative factor of abnormal extracellular matrices, vascular calcification with arterial stiffness, atherogenesis, and high pulse pressure [71,87]. TIMP-1 has been shown to exert a similar role. In CKD patients, TIMP-1 serum concentrations were significantly increased from Stage I–III to IV–V [88]. Moreover, TIMP-1 has been found to be a predictor of the new onset of CKD and heart failure, regardless of age, gender, and biomarkers of systemic inflammation (c-reactive protein and brain natriuretic peptide) [40]. TIMP-1 is also increased in patients with PAD, with ischemic muscle being a possible source for this enzyme [12]. Interestingly, an association between TIMP-1 levels and TGF-β was described in young patients with CKD, suggesting that this factor may be involved in the pathogenesis of inflammation and CV damage even at an early stage of the disease [89].

A further crucial role in the link between metalloproteinases, CKD, and PVD is indicated by NGAL. It has been shown that NGAL levels are increased in atherosclerosis and chronic inflammatory processes, and in particular, are overexpressed in atherosclerotic plaques which are vulnerable to rupture [90]. Moreover, NGAL circulating levels have been found to increase in patients with CKD, being directly related to diabetic status and inversely to the eGFR levels [91]. From a prognostic perspective, in both CKD and general populations, plasma NGAL was a significant predictor of CV fatal and nonfatal events, including PVD [92,93]. These findings were globally reinforced by the evidence that plasma and tissue NGAL expression contribute to the pathogenesis and severity of central and peripheral aneurismal disease [94]. In this context, it has been proposed that NGAL reflects the leukocyte-mediated inflammatory response, with higher values being associated with weakness of the vascular wall and impaired vascular damage [95]. (2) CKD is characterized by a persistent low-grade inflammation with the production of pro-inflammatory cytokines, and MMP activity has been implicated in these pathways. The uremic milieu may also induce oxidative stress and recurrences in thrombotic events and infections, as well as impairment in arterial stiffening and calcification of both the intima and media of the arterial wall [55]. In the pathogenesis of all these processes, a crucial step is represented by ECM remodeling. MMPs are directly involved in ECM processes in almost all tissues. (3) In patients with already diagnosed CKD, serum levels of MMPs have been found to be significantly associated with the degree of proteinuria. This means that MMPs may be directly implicated in the development of glomerular or tubular damage typical of a wide spectrum of kidney diseases. The presence of proteinuria is a strong predictor of CV events (and PVD as well), and is recognized as a marker of endothelial systemic damage. Moreover, its prognostic role is even stronger than that attributed to the traditional CV risk factors in CKD patients (i.e., Framingham risk factors) [7,8,48]. Indeed, MMPs are detected and elevated in human kidneys with nephritic syndrome, which is a high-risk condition with severe proteinuria [51,96]. (4) It should not be excluded that MMPs play a predictive other than a prognostic role in patients with CKD or PVD. This means that the reduction of MMPs following a selective treatment could be associated with a better prognosis in these patients. Recently, several therapeutic agents that modulate the function of MMPs have been developed. The tetracycline antibiotic doxycycline and the nonselective inhibitors of MMPs, Batimastat and Marimastat, have been shown to cut vascular tissue remodeling by reducing MMP activity [97,98]. However, larger clinical studies are needed to generalize and translate these effects to clinical application. Moreover, the reduction in proteinuria that followed the use of doxycycline was associated with a reduction in the glomerular expression of MMP [58]. Also, the Renin-Angiotensin-Aldosterone system inhibitors (RAAS-I), that are currently considered the most effective renoprotective drugs against the risk of ESKD and CV events in CKD patients, have been shown to decrease the MMPs levels [99,100,101]. However, all these important pieces of evidence have led to the hypothesis that MMPs could be one of the causal links between CKD and CV risk, including PVD. (5) In the era of precision medicine, the need to ameliorate prognoses in patients with chronic diseases is currently increasing [102]. To this end, a number of clinical trials evaluating the beneficial effect of new drugs on cardiovascular (including peripheral vascular disease) and renal outcomes have been started [103]. Interventions varied between studies, with the effect of blood pressure lowering drugs, albuminuria lowering agents, diuretics, and endothelin receptor antagonist being tested. Results from these first studies increased the interest in a relatively new drug class, Sodium–glucose cotransporter 2 (SGLT-2) inhibitors. SGLT2-Is reduce the reabsorption of glucose from the proximal tubules of the kidney. The SGLT2-Is canagliflozin and empagliflozin reduced the risk of developing fatal and nonfatal CV events in previous trials, with this effect being associated with a reduction of albuminuria in the first 3 to 6 months of treatment [104,105]. However, an interesting recent analysis of human proximal tubular epithelial cells showed that empagliflozin is also able to reverse the renal suppression of Reversion Inducing Cysteine Rich Protein with Kazal Motifs (RECK), a membrane anchored endogenous MMP inhibitor whose expression is induced by hyperglycemia in animal and human models of diabetic kidney disease [106]. Interestingly, empagliflozin reduced the epithelial-to-mesenchymal transition, a mechanism that is strictly linked to MMPs activity and that anticipates kidney fibrosis, by directly reducing migration of tubular cells and expression of pro-inflammatory mediators (TRAF3IP2, NF-κB, p38MAPK, miR-21, and IL-1β, IL-6, TNF-α, and MCP-1). This means that SGLT2-Is may exert a protective effect on renal tubules that is independent from the albuminuria reduction. Thus, new large intervention studies evaluating the effect of drugs that interfere with MMPs activity are expected in the future to evaluate their impact on prognoses of albuminuric and nonalbuminuric CKD patients. This would be particularly important considering that nonalbuminuric CKD patients are growing in prevalence as referred patients in nephrology clinics [47,107]. In the context of intervention studies, MMPs may play a role as “predictive” biomarkers, since their assessment could identify a population of patients which is more likely to benefit from a specific drug. This point is crucial, since the development and optimization of new treatments that reduce CV risk in CKD patients may help to overcome the variability in response to the old standard treatments [108].

In addition to clinical trials perspective, further efforts are needed in the context of observational studies and risk stratification. Indeed, risk stratification of CKD patients may improve prevention strategies, acting to slow down CKD progression and reduce the high residual risk in CKD patients. In observational research, it may be useful to evaluate the potential role of MMPs as “prognostic” biomarkers used to identify the likelihood of a patient to develop a clinical outcome, regardless of treatment (i.e., under the standard of care). Such a measure may improve physicians’ abilities to identify patients with poor prognoses [109]. One approach involves the discovery of novel biomarkers that may add proper prognostic information on top of already known risk factors [46,107,110,111,112,113]. Investigators from the Steno Diabetes Center, a prospective cohort that contributed to the comprehension of prognosis of diabetic patients with or without CKD, showed that MMP-1 and MMP-2 are associated with an increased risk of CV events and CV mortality, regardless of age, gender, eGFR, and albuminuria [114]. A similar effort in improving risk stratification is ongoing in the case of PAD management. It has indeed been demonstrated that patients suffering from PAD are exposed to a higher risk of CV events than those with coronary heart disease [115,116]. Hence, ameliorating prognoses of these patients is mandatory and urgent. A previous recent analysis showed that variations in the serum levels of MMPs before and after lower limb surgical revascularization in patients with critical limb ischemia are significantly associated with the subsequent outcome (major amputations or death). From our research, we can also assert that measuring the serum levels of MMPs could be even more useful in patients with CKD, PVD, or both conditions, as they potentiate the risk of progression for these conditions.

## Figures and Tables

**Table 1:** Summary of main paper results.

	Key-Messages
**MMPs and CKD**	The metalloproteinases (MMPs) are associated with kidney damage. Increased levels of MMP-2 and MMP-9 mediate the deposition of extracellular matrix (ECM) in the glomerular cells while inducing the loss of cellular junctions, and start epithelial-mesenchymal transition (EMT) in the tubular cells. These processes result in tubular atrophy and fibrosis [52,70]. In the pathogenesis of renal damage, a crucial role is played by the downregulation of MMP-1 and the overexpression of TIMP-1, MMP-2, MMP-7, MMP-9, and neutrophil gelatinase-associated lipocalin (NGAL). These molecules are involved in the inflammatory process and in all phases of renal fibrosis, which, taken together, lead to a progressive decline in renal function. Furthermore, MMP-3 could also promote EMT [32,37,38,39]. The cardiovascular risk among patients with Chronic Kidney Disease (CKD) is not trivial. These patients experience a higher rate of cardiovascular events over time than kidney disease progression [5,6,7]. In CKD patients, the combination of uremic milieu, oxidative stress, and imbalance in pro-inflammatory substances, such as MMPs and metalloproteinase tissue inhibitors (TIMPs), amplifies the atherosclerotic process, arterial stiffness and vascular calcification. MMP-2 and MMP-9 have been implicated in carotid Intima-Media Thickness (cIMT) and plaque instability. Moreover, increased levels of TIMP-1 have been associated with a higher risk of CHF [49,50,51,52].
**MMPs and PVD**	MMPs play a role in the pathogenesis of peripheral vascular disease. It has been demonstrated that TIMP-1, MMP-1, -2, -3, -9, and -14 favor the progression of atherosclerotic plaques. Increased levels of MMP-2, -3, and -9 are involved in the weakening of the aneurysm wall. In relation to varicose veins, the expression of MMP-1, -2, -3, -7, and -9 is increased. Therefore, the amount of these MMPs is related to a major risk of amputation or death in patients who underwent lower extremity bypass, making that parameter a good predictor of poor prognosis [27,28,29,30,31,59]. Circulating levels of MMP-2 and MMP-9 were found in patients with acute and chronic lower limb ischemia. These MMPs induce the release of transforming growth factor beta (TGF-β). TGF-β affects the balance between endothelial cells and smooth muscle cells [71].
